# Enhancing agroecosystem productivity with woody perennials in semi-arid West Africa. A meta-analysis

**DOI:** 10.1007/s13593-018-0533-3

**Published:** 2018-10-23

**Authors:** Georges F. Félix, Johannes M. S. Scholberg, Cathy Clermont-Dauphin, Laurent Cournac, Pablo Tittonell

**Affiliations:** 1 Farming Systems Ecology, Wageningen University & Research, Droevendaalsesteeg 1, 6708 PB Wageningen, The Netherlands; 2 Eco&Sols, Univ Montpellier, CIRAD, INRA, IRD, Montpellier SupAgro, IRD, Campus SupAgro, Bâtiment 12, 2 place Viala, 34060 Montpellier Cedex 2, France; 3 LMI IESOL, Centre IRD-ISRA de Bel Air, BP1386, CP18524 Dakar, Senegal; 4 Natural Resources and Environment Program, CONICET-INTA, Modesta Victoria, 4450 San Carlos de Bariloche, Río Negro Argentina

**Keywords:** Agroforestry, Mulch, Sahel, Shrub-crop associations, Woody amendments

## Abstract

Soil degradation in semi-arid West Africa can be reversed through an intensified application of organic matter, especially on coarse soils. Woody perennials have been promoted in the region to secure organic matter sources and improve soil productive capacity, yet the mechanisms by which perennials provide benefits to soils and crops remain poorly understood, and no effective, generalizable agronomic recommendations exist. Here, we reviewed the effects of trees and shrubs on soil properties and on crop yields in semi-arid West Africa (< 1000 mm year^−1^). Specific objectives of this meta-analysis were to (i) describe and (ii) quantify the effects of the presence of woody perennials and of ramial wood amendments on crop productivity and soil characteristics, and (iii) identify general recommendations on the integration of perennials with crops. An iterative keyword search was conducted to gather relevant literature. The search string consisted of four parts: source, practice, responses, and countries of interest. In total, 26 references on agroforestry parklands and 21 on woody amendments were included in the meta-database (314 entries, 155 for parklands, and 159 for ramial wood). We show that (1) the presence of shrubs and trees on agricultural fields had an overall positive but variable effect on soil total C (i.e. + 20 to 75%); (2) millet and sorghum yields were often higher in the presence of shrubs (− 25 to + 120%); (3) more variability was observed in the presence of trees (− 100 to + 200%); and (4) the use of shrub- and tree-based ramial wood resulted in equal or higher cereal yields as compared to the control (− 30 to + 100%). Upscaling the use of biodiversity-driven processes in farming systems of West Africa may provide benefits to overall ecosystems, but species’ choice and trade-offs perceived at the farm level, including labour management and low ramial wood availability, should be addressed through future research.

## Introduction

Agricultural-based economies prevail in semi-arid West Africa (SWA), yet harsh growing conditions such as extreme temperatures, erratic and low rainfall, and strong erosive winds on already nutrient-depleted soils typically result in very low productivity or at times complete crop failure (Diarisso et al. 2015). Traditional agricultural areas near population centres are lost due to urban development, and agriculture is pushed onto both marginal land and forested areas (Reij et al. 2005; Doso Jnr 2014). The human population of West Africa (WA) has increased from 106 million in 1970 to 305 million in 2010, and the current annual growth rate of 2.78% is one of the highest in the world (World Bank 2016). Population growth in rural areas intensifies pressure on land resources for subsistence farming (Andrieu et al. 2015) by limiting the traditional practice of keeping fallow periods to restore soil fertility (Bonetti and Jouve 1999).

Continuously cultivated crop fields and reduced fallow periods are short-chained soil restoration processes which do not compensate for the decline in soil organic matter in most soils used for agriculture in the region (Kintché et al. 2015). Especially when coinciding with poor soil management and/or more extreme weather conditions, acute soil degradation may be the final outcome. This vicious circle of soil fertility decline (Lal 2008) often results in the formation of non-responsive, degraded soils (Tittonell and Giller 2013), represented on almost 600,000 km^2^ of WA, half of which are featured by intensively weathered and inherently infertile soils in semi-arid environments (Bai et al. 2008).

Concentrated efforts to reverse soil degradation by restoring soil productive capacity of marginal agricultural lands are essential to feed and sustain the livelihoods of a continuously growing population (Tittonell 2016). SWA soils are coarse and intensively weathered, and this translates into limited capacity to store and protect soil organic matter (Bationo et al. 2007). Organic matter accumulation in the soil can be achieved by increasing C-rich inputs and reducing C outputs from the soil. Organic inputs or amendments such as animal manure or crop residue mulches play a key role in soil restoration. Sources of organic matter in SWA landscapes are not so abundant and rather scattered (Gijsbers et al. 1994; Sop et al. 2011). Therefore, novel options to secure organic matter inputs to soil are required.

Crop residues are preferentially used to feed domestic ruminants or as fuel (de Ridder et al. 2004; Giller et al. 2009), so woody vegetation can provide an in situ source of leaf and branch biomass for soil amendment (Bayala et al. 2003; Diack et al. 2010; Dossa et al. 2012a; Diedhiou-Sall et al. 2013; Dossa et al. 2013; Yélémou et al. 2014). Ramial wood (RW) amendment availability relies on existing tree and shrub vegetation. The mechanisms by which woody amendments may provide benefits to soils and crops remain poorly understood, and no effective agronomic recommendations on the use of leaf and branch material currently exist in the SWA context (Bayala et al. 2003; Barthès et al. 2015; Félix et al. 2018).

The use of leaf and branch litter as soil amendments has been extensively evaluated in Canada and in temperate areas of Europe (Barthès et al. 2010) where the application of large amounts of ramial chipped wood mulch (RCW; in French *bois raméal fragmenté*, BRF) improved soil structure, enhanced fungal activity (i.e. *Basidiomycetes*), and increased crop yields (i.e. + 30% in potatoes and + 300% in strawberries) (Lemieux 2001).

In semi-arid West Africa (SWA), the question remains as to whether biomass derived from trees and shrubs can provide an adequate soil amendment to improve soil quality and crop productivity, in a way that is sustainable and accessible to farmers. A systematic literature review was conducted to compile relevant case studies in SWA and elucidate the effects and corresponding mechanisms by which the woody perennials and the biomass they produce may help to regenerate or enhance soil productive capacity. Specific objectives of this meta-analysis were (i) to describe interactions between agroecosystem components, (ii) to quantify the effects of the presence of woody perennials and the use of ramial wood amendments on crop productivity and soil characteristics, and (iii) to identify general recommendations on the integration of perennials with crops. Materials and methods for the selection of studies and indicators examined in this meta-analysis are described in the next section, followed by the results and discussion on the effects, feasibility, and trade-offs of scaling up the use of woody perennials in agricultural landscapes of SWA.

## Materials and methods

### Geographical zone and farming systems

**Fig. 1:**
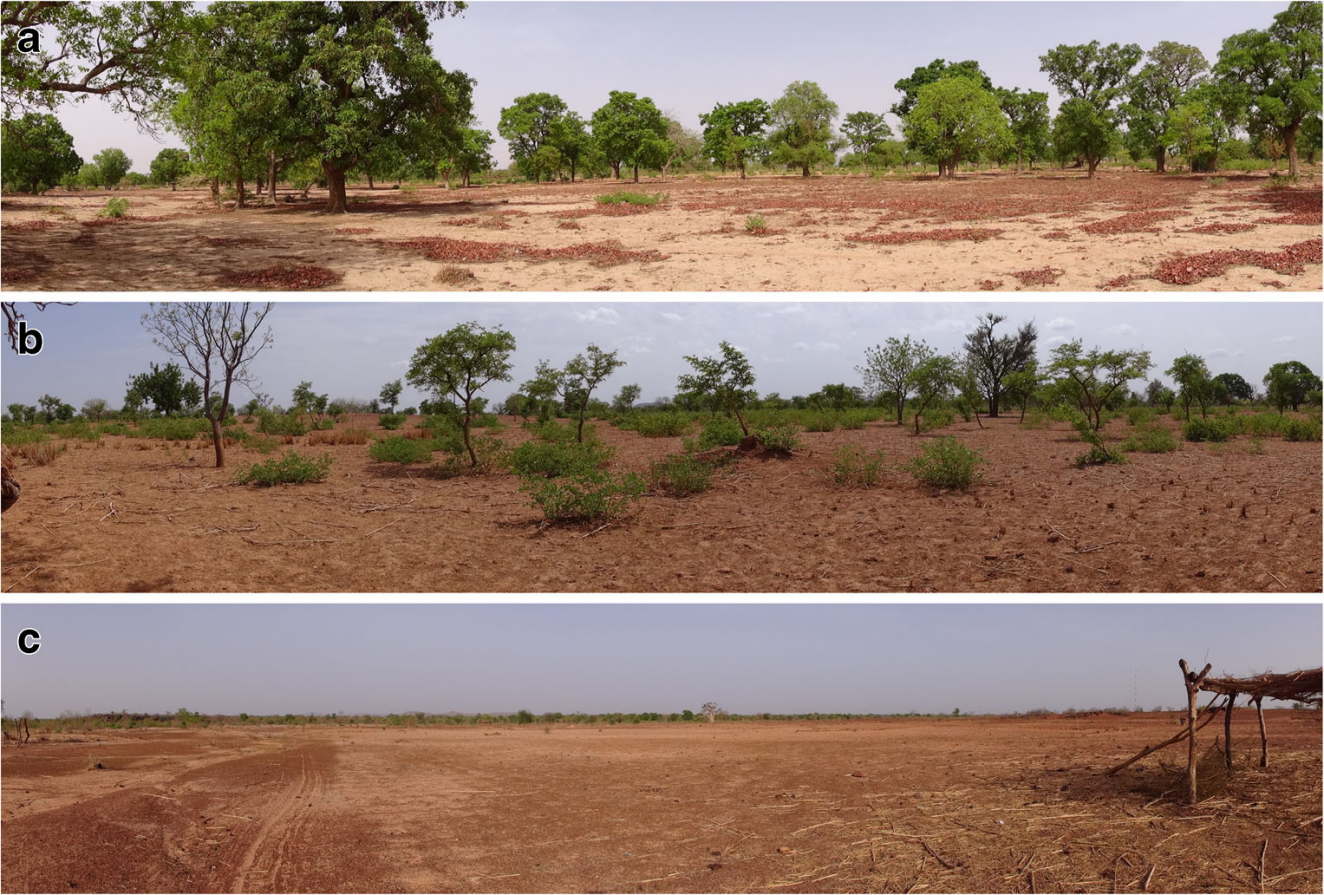
Remnant woody perennials during dry season drive soil heterogeneity of agricultural fields via above- and belowground interactions. Shea nut trees provide abundant leaf litter and support nutrient recycling (**a**). *Piliostigma* shrubs are managed as an off-season micro-fallowing system (**b**). Finally, bare fields will usually require more external inputs, including livestock manure, chemical fertiliser, or crop residues, to support nutrient balances (**c**). Photos: G. Félix, Yilou, Burkina Faso

**Fig. 2:**
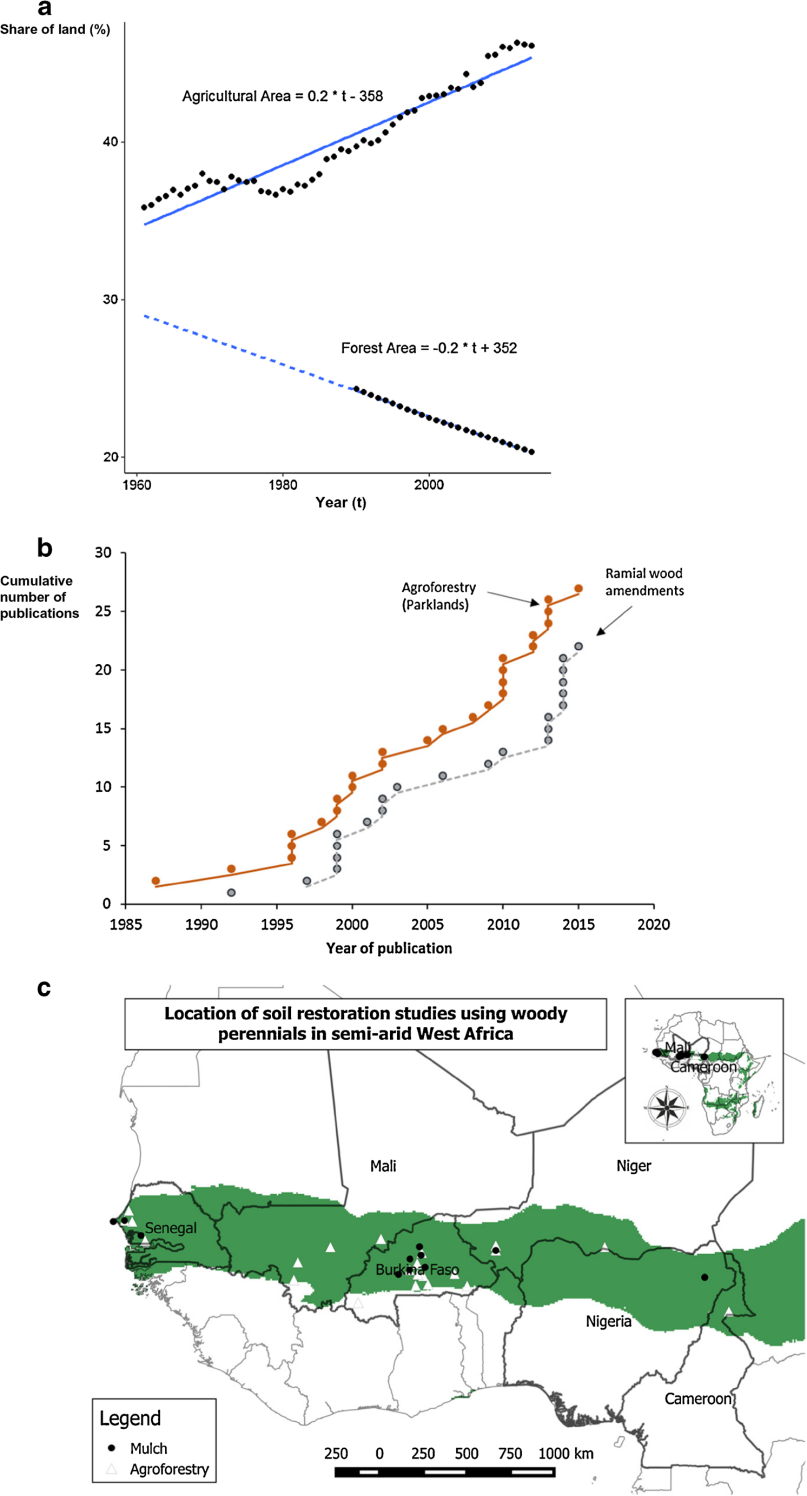
Agricultural area (cropland) in West African countries increases at the expense of forest land; data source: FAOstats—Land Use Indicators, average share of land (%) for Benin, Burkina Faso, The Gambia, Mali, Mauritania, Niger, Nigeria, and Senegal (**a**). Cumulative number of publications on the impacts of agroforestry systems and woody mulch application on soil fertility and crop yields in semi-arid West Africa between 1987 and 2015 (**b**). Map of the region considered in this literature review (**c**)

Agricultural landscapes of West Africa (WA) are heterogeneous (Fig. 1; Zorom et al. 2013), ranging from deserts and semi-arid ecosystems to moist savannah, humid forests, and swamps (Jalloh et al. 2012). Increasing rainfall from north to south drives an increasing complexity of land use in WA, including agropastoral systems in the driest areas and cropping or mixed farming systems in the wetter zones (Table 1). Agricultural areas have increased at the expense of forest areas (Fig. 2a). The geographical range of this meta-analysis covers SWA, including part of Senegal, The Gambia, Mauritania, Mali, Burkina Faso, Northern Benin, Niger, Northern Nigeria, and Northern Cameroon (Fig. 2c). This semi-arid “belt” also corresponds partly to the location of the “Great Green Wall” project, an international effort to counter the advancement of desertification (Dia and Duponnois 2012). The region of study features a range of 300–1000 mm annual rainfall, concentrated in a single and relatively short period (unimodal rainfall pattern of 60 to 120 days) each year (Dixon et al. 2001; West et al. 2008). In the absence of irrigation, it is possible to successfully cultivate millet (*Pennisetum glaucum*) and sorghum (*Sorghum bicolor*), as well as maize (*Zea mays*) towards the wetter southern parts of the rainfall gradient. Millet and sorghum crop yields (grain and biomass) were selected as indicators for our quantitative analysis.

**Table 1:** Sub-regions, ecosystems, and farming systems of West Africa

Climatic sub-region	Months of rain	Annual rainfall (mm)	Average annual temperature (°C)	Land cover	Dominant farming system
Saharo-Sahelian	1–2	250–500	24.4–28.5	Steppe with thorny bushes and annual grasses	Agropastoral—millet farming system. Mostly pastoral (transhumant herding) with pockets of subsistence farming based on millet, sorghum, and cowpea
Sahelian	1–3	300–550	24.4–28.5	Steppe with thorny bushes and annual grasses	Agropastoral—millet/sorghum farming system. Mostly pastoral (transhumant herding) with pockets of subsistence farming based on millet, sorghum, and cowpea
Sudano-Sahelian	2–3	350–600	23.7–25	Steppe with *Combretum* and annual grasses	Agropastoral—millet/sorghum farming system. Combination of transhumant herding and sedentary agropastoral agriculture. Subsistence farming based on millet, sorghum, and cowpea
Sudanian	3–4	500–900	23.7–25	Savannahs with trees (*Balanites aegyptiaca*, *Acacia* spp) and shrubs	Cereal-root crop farming system. Mix of agricultural and agropastoral activities. Sedentary farming dominant, including sedentary village stock raising and permanent cropping of sorghum, millet, cowpea, cassava, cotton, and groundnut, with transhumant pastoralism during the dry season
Sudano-Guinean	4–5	750–1200	24.5–28.8	Savannah with trees or shrubs, sparse forests	Cereal-root crop farming system. Agricultural area characterised by perennial crops (mangos, citrus, cashew, etc.), cotton, cassava, yam, and cereals (sorghum, millet, and maize). Sedentary village stock bull rearing, with transhumant grazing during the dry season

Adapted from Arbonnier 2000; Dixon et al. 2001

**Fig. 3:**
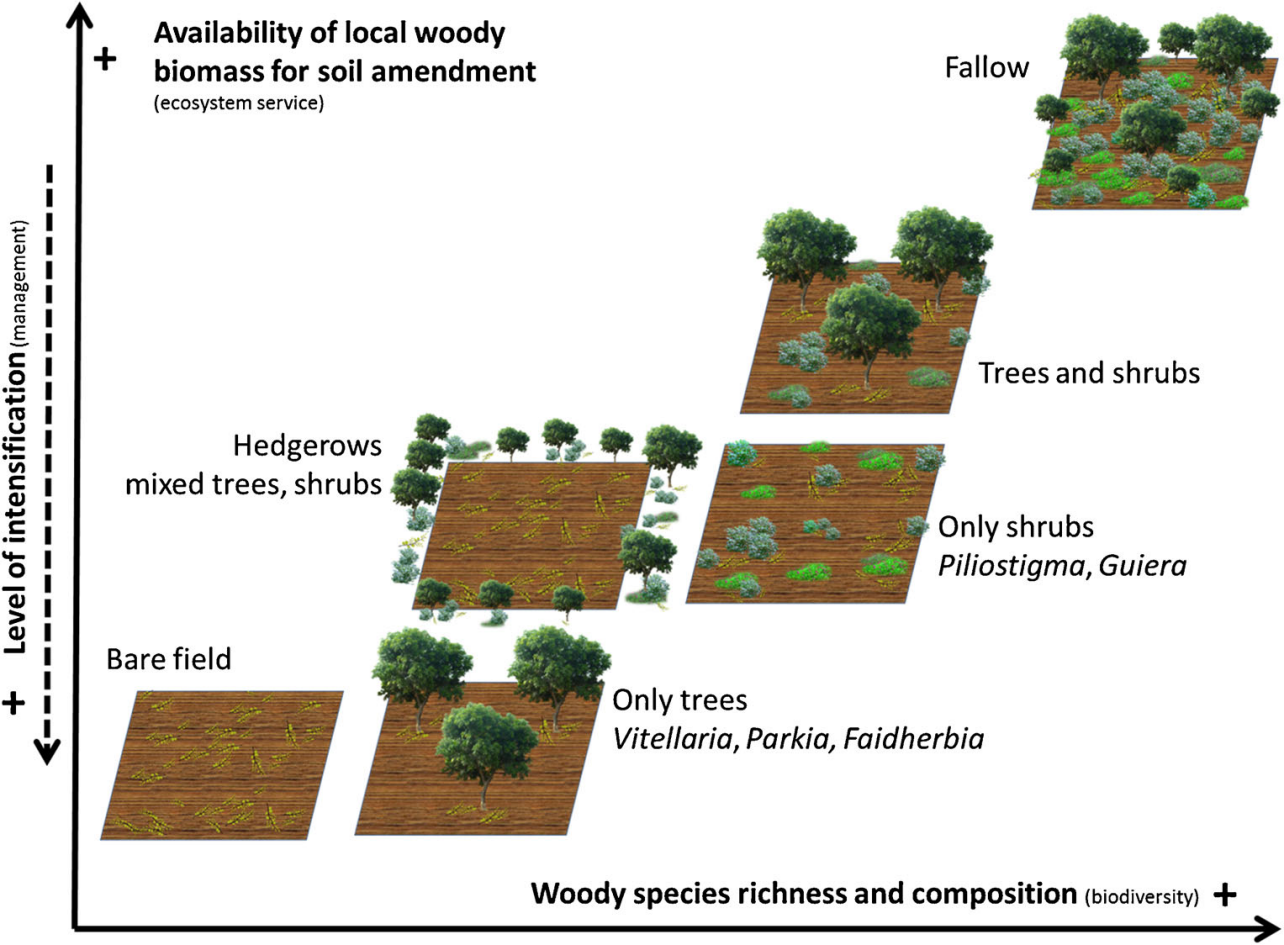
Conceptual agroecosystem designs for soil management with woody perennials, defined by local availability of woody species for soil amendment as a function of woody species diversity at field level. Intensification level refers to the degree of transformation of the original landscape (i.e. fallow)

Landscapes across SWA include trees and woody shrubs growing in or around the cropping fields, often reproducing the structure of an open savannah, or hereafter “parklands” (Boffa 1999). Trees are present in hedgerows or interspaced between crop plants within cropping fields, sometimes mixed with woody shrubs (Fig. 3). Potential in situ amounts of woody material available for soil amendment depend largely on agroecosystem design and woody perennial integration at the cropping system level (Sop and Oldeland 2013; Feur 2014; Cheriere 2015).

### Selection of criteria used for the literature review

A three-step procedure was employed to select papers related to woody perennial-based cropping systems in SWA. The first step was an iterative keyword search to capture relevant literature related to the effects of woody perennial species and associated leaf and branch material on restoration of soil productive capacity in semi-arid cropping systems. The search string consisted of four parts: the first search terms related to the source of the woody perennial (“tree” or “shrub”), the second to the management practice (“mulch” or “(inter)cropping”), the third to the response variables (e.g. soil properties and crop productivity), and the last one related to the specific environmental context (specific countries within SWA). The final combination of search keywords (Table 2) yielded 91 results in Scopus (based on titles, abstracts, and keywords) and 267 results on ISI Web of Science (based on topic).

**Table 2:** Search terms and strings used during the literature retrieval

Search terms related to	Keywords	Scopus	Web of Science™ core collection
*Source*	(shrub* OR bush* OR wood* OR branch* OR leaf* OR tree* OR biomass)	91	267
*Practice*	AND (mulch* OR *crop*)		
*Response*	AND (soil fertility OR soil restoration OR soil quality OR productivity OR yield*)		
*Context*	AND (semi*arid OR arid OR sudan* OR soudan* OR sahel* OR senegal OR gambia OR mali OR mauritania OR burkina faso OR niger OR nigeria OR benin)		

The second step consisted of manual screening of titles and abstracts of citations using the following criteria:

- Studies that were conducted in the defined environmental context (rainfall < 1000 mm year^−1^ in semi-arid agroecosystems of Sudano-Sahelian Africa, including Senegal, The Gambia, Mauritania, Mali, Burkina Faso, Northern Benin, Niger, Nigeria, and Northern Cameroon).
- Studies that included woody amendments as a management practice (surface-mulched or buried branches and/or leaves, but not biochar), and describing local uses of woody residues, or the presence of woody shrub or tree vegetation in farmers’ fields, and reported effects on soil quality and/or crop productivity.
- Studies conducted on either farmers’ fields or experimental stations were typically included with corresponding field data; pure modelling results were excluded.
- Literature reviews were excluded from the meta-analysis but were used to cross-check data, methods, and references.

From the total of 91 references of the Scopus search, 71 titles and abstracts did not meet one or more of the above-listed criteria. Only 15% of the references were included in our database; these had clearly described treatments and experimental results. Additional references retrieved from ISI Web-of-Knowledge often concerned areas outside our study region (China or other parts of Africa). Thus, we only included those that were also present from the Scopus search and those relevant for the construction of our database (contributing with six additional references).

During a third step, papers were reviewed in full detail and key parameters and figures regarding production environment, management practices, and response variables were compiled in a spreadsheet, checking for consistency in terms of scales and units and recalculating when necessary to obtain a common measure (and unit) for each target variable.

This information was complemented by grey literature, including one BSc thesis, three MSc theses, four PhD theses, and four reports by international organisations. Most of this literature was retrieved from the Wageningen University Library, The Netherlands, and from the library of *Centre d’Information Commun sur la Recherche et le Développment* (CICRD) located at the IRD/CIRAD campus in Ouagadougou, Burkina Faso. Supplementary data presented in the online version of a review paper by Bayala et al. (2014) were added to our dataset to analyse effects of trees in parklands, contributing 16 additional references. In total, 47 references (Table 3) were included in the meta-database that was eventually analysed (26 references on parklands, 21 on ramial wood amendment applications).

**Table 3:** List of publications, the study locations within semi-arid West Africa, the woody species (or RW source), and the crop of study

Authors	Source	Country of study	Woody species (or source)	Crop of study	Yield data rows
Agroforestry					
Bayala (2002)	PhD thesis	Burkina Faso	*Parkia biglobosa*, *Vitellaria paradoxa*	Sorghum, millet	8
Boffa (1999)	*Agroforestry Systems*	Burkina Faso	*V. paradoxa*	Sorghum	2
Charreau and Vidal (1965)	*Agronomie Tropicale*	Senegal	*Faidherbia albida*	Millet	2
Depommier et al. (1992)	Proceedings	Burkina Faso	*F. albida*	Sorghum	6
Diakhaté et al. (2013)	*European Journal of Agronomy*	Senegal	*Piliostigma reticulatum*	Millet	1
Dibloni et al. (1999)	Report	Burkina Faso	*Albizzia lebbeck*, *F. albida*, *Prosopis africana*	Sorghum	18
Dossa et al. (2012)	*Agronomy Journal*	Senegal	*Guiera senegalensis*	Groundnut, millet	7
Dossa et al. (2013)	*Agronomy Journal*	Senegal	*P. reticulatum*	Groundnut, millet	7
Dan Lamso et al. (2016)	*International Journal of Biological and Chemical Sciences*	Niger	*G. senegalensis*, *Hyphaene thebaica*	Millet	4
Harmand et al. (1996)	*Cahiers Scientifiques*	Cameroon	*F. albida*	Sorghum	4
Jonsson et al. (1999)	*Experimental Agriculture*	Burkina Faso	*P. biglobosa*, *V. paradoxa*	Millet	4
Kizito et al. (2007)	*Journal of Arid Environments*	Senegal	*P. reticulatum*, *G. senegalensis*	Millet	2
Louppe et al. (1996)	*Cahiers Scientifiques*	Senegal	*F. albida*	Groundnut, millet	18
Maiga (1987)	MSc thesis	Burkina Faso	*F. albida*, *P. biglobosa*, *V. paradoxa*	Millet, sorghum	6
Oliver et al. (1996)	*Cahiers Scientifiques*	Burkina Faso	*F. albida*	Sorghum	6
Pouliot et al. (2012)	*Agroforestry Systems*	Burkina Faso	*P. biglobosa*	Chili pepper, eggplant, millet, taro	6
Sanou et al. (2011)	PhD thesis	Burkina Faso	*Adansonia digitata*, *P. biglobosa*	Millet, taro	4
Sidibe (2010)	PhD thesis	Mali	*Tamarindus indica*	Eggplant local, sorghum	6
Wezel (2000)	*Agroforestry Systems*	Niger	*G. senegalensis*	Millet	8
Wilson et al. (1998)	*Experimental Agriculture*	Burkina Faso	*P. biglobosa*	Sorghum	2
Yaméogo (2008)	PhD thesis	Burkina Faso	*Borassus akeassii*, *F. albida*	Maize	2
Yélémou et al. (2013)	Tropicultura	Burkina Faso	*P. reticulatum*	Sorghum	18
Zomboudré et al. (2005)	*Biotechnol. Agron. Soc. Environ.*	Burkina Faso	*V. paradoxa*	Maize	4
Ramial wood (buried)					
Barthès et al. (2015)	*Agroforestry Systems*	Burkina Faso	*P. reticulatum*	Sorghum	12
Roose et al. (1999)	*Arid Soil Research and Rehabilitation*	Burkina Faso	*Azadirachta indica*	Sorghum	8
Samba (2001)	*Annals of Forest Science*	Senegal	*Cordyla pinnata*	Millet, peanut	9
Soumare et al. (2002)	*Biological Agriculture and Horticulture*	Senegal	*Casuarina equisetifolia*	Tomato	6
Ramial wood (mulch)					
Barthès et al. (2015)	*Agroforestry Systems*	Burkina Faso	*P. reticulatum*	Sorghum	6
Bayala et al. (2003)	*Arid Land Research and Management*	Burkina Faso	*P. biglobosa*, *V. paradoxa*	Millet	16
Belliard (2014)	BSc thesis	Burkina Faso	*P. reticulatum*	Cowpea, sorghum	6
Chiroma et al. (2006)	*Experimental Agriculture*	Nigeria	Wood waste	Sorghum	12
Ibrahim et al. (2015)	*Nutrient Cycling in Agroecosystems*	Niger	*Acacia tumida*	Millet	12
Mando and Stroosnijder (1999)	*Soil Use and Management*	Burkina Faso	*Pterocarpus lucens*	Natural vegetation	8
Ouédraogo (2014)	MSc thesis	Burkina Faso	*P. reticulatum*	Cowpea	6
Salau et al. (1992)	*Soil & Tillage Research*	Nigeria	Wood shavings	Plantain	1
Sanou (2015)	MSc thesis	Burkina Faso	*P. reticulatum*	Sorghum	6
Shiyam et al. (2011)	*World Journal of Agricultural Sciences*	Nigeria	Sawdust mulch	Cocoyam, plantain	8
Somé (2014)	MSc thesis	Burkina Faso	*P. reticulatum*	Sorghum	6
Tilander and Bonzi (1997)	*Plant and Soil*	Burkina Faso	*Acacia holocericea*, *A. indica*	Sorghum	6
Wezel and Böcker (1999)	*Soil & Tillage Research*	Niger	*G. senegalensis*	Millet	4
Yélémou et al. (2014)	*Journal of Plant Studies*	Burkina Faso	*P. reticulatum*	Sorghum	12
Yossi et al. (2002)	Report	Mali	*P. africana*	Millet	2

In the selected studies, experimental treatments were located under tree canopy or, in the case of shrubs, within the vicinity of their canopy projection. Control data were from outside the area of canopy influence. Data concerning use of ramial wood (RW) amendments (mulched or buried) were considered with control treatments that did not apply RW as soil amendment.

### Search metrics and overview

**Fig. 4:**
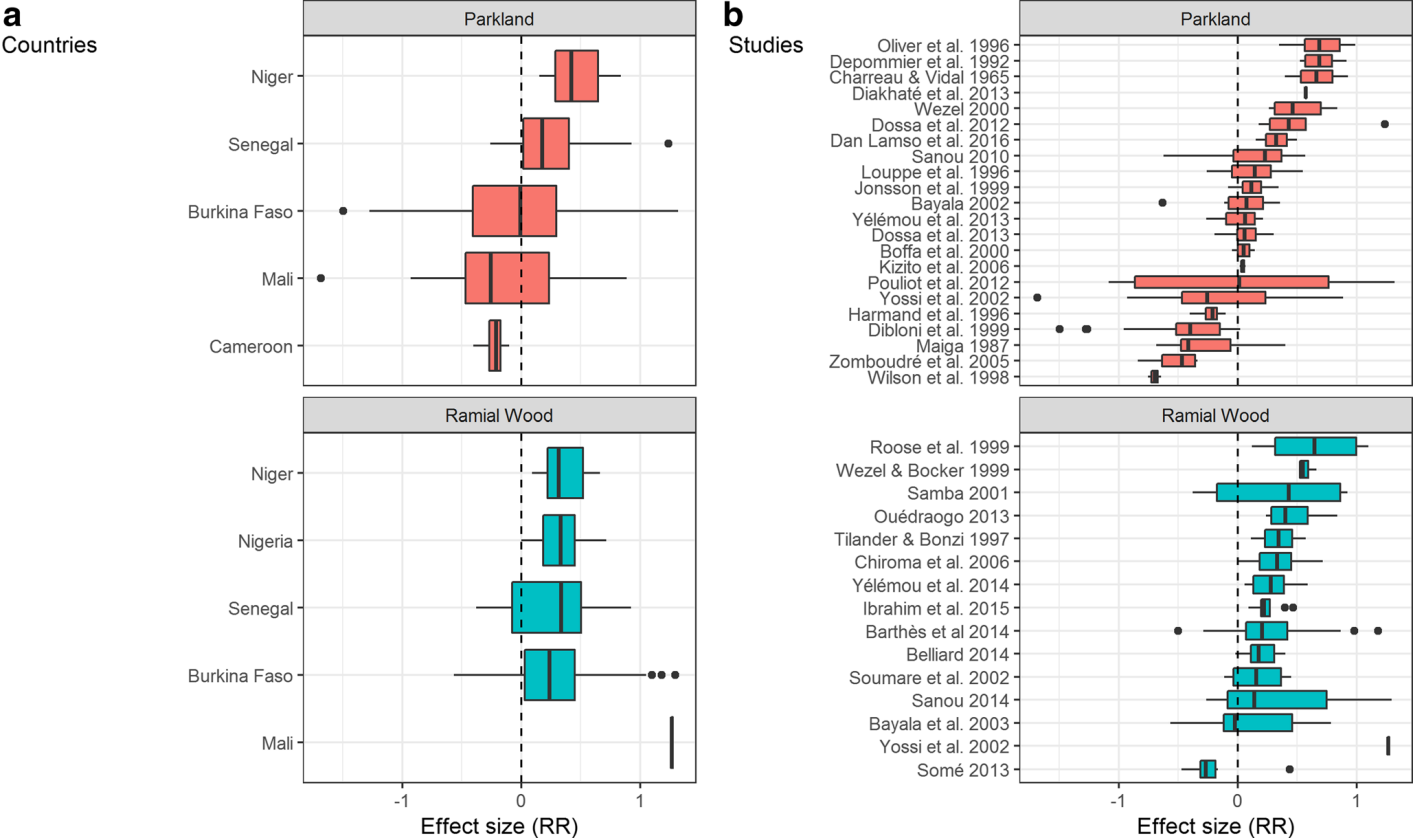
Variability of yield effect size per country (**a**) and per study (**b**)

The earliest publication retrieved in our search on the effect of parkland agroforestry on soil and crop productivity was published in 1965 with the example of *Faidherbia albida* in Senegal (Charreau and Vidal 1965). The oldest publication retrieved on ramial wood amendments in SWA is dated 1997 and was linked to keyword “agroforestry mulches” (Tilander and Bonzi 1997). The number of publications retrieved that reported on the use of biomass from woody perennials for soil amendment, whether through interactions in agroforestry parklands or as biomass transfer (cut and carry), plus the corresponding impacts on soil quality and crop yields in SWA, increased from less than ten prior to 1996 to about 20 between 1997 and 2005; another 20 publications appeared between 2005 and 2015 (Fig. 2b; Table 3). Most of the studies retrieved originated from Burkina Faso and Senegal, while Cameroon, Mali, Niger, and Nigeria were represented in a limited number of publications (Fig. 4a).

No homogenised term for ramial wood amendment practice exists. As a consequence, across the literature, the following terms were identified as being similar or closely related to ramial wood amendments:

- Agroforestry mulches (Tilander and Bonzi 1997)
- Prunings (Bayala et al. 2003)
- Leaf mulch (Bayala et al. 2005)
- Woody perennial leaf biomass (Yélémou et al. 2014)
- Woody biomass (Gruenewald et al. 2007; Debela et al. 2011)
- Wood shavings (Chiroma et al. 2006; Gajalakshmi and Abbasi 2008)
- Wood waste (Bulmer et al. 2007; Andry et al. 2011)
- Woody debris (Brown and Naeth 2014)
- Wood (Bonanomi et al. 2014)
- Native agroforestry plant residues (Iyamuremye et al. 2000)
- Native shrub residues (Dossa et al. 2009)
- Branches of indigenous shrub (Wezel and Böcker 1999)
- Shrub material (Chapuis-Lardy et al. 2015)
- Ramial chipped wood (Gómez 1997; Robert et al. 2014)
- Ramial wood amendments (Barthès et al. 2015; Félix et al. 2018)
- Chopped twig wood (Aman et al. 1996)
- *Rameaux ligneux* (Kabré 2010)
- *Bois raméal* (Barthès et al. 2010)
- *Bois raméal fragmenté* (Zongo 2009; Ba et al. 2014; Somé 2014)

A total of 19 woody species were documented in the literature reviewed (Table 4). *Faidherbia albida* (*n* = 48) gathered the most entries in our agroforestry parkland database, followed by the tree species *Parkia biglobosa* (*n* = 18) and *Vitellaria paradoxa* (*n* = 17), and the shrub species *Piliostigma reticulatum* (*n* = 25) and *Guiera senegalensis* (*n* = 21). *P. reticulatum* (*n* = 52) and *G. senegalensis* (*n* = 14) were the most represented species in experimental studies addressing the use of woody and leafy mulches as soil amendments in our database.

**Table 4:** Botanical families and Latin and English names of woody perennial species included in this meta-analysis

Botanical family	Latin name	English name
Anacardiaceae	*Sclerocarya birrea* (A. Rich.) Hochst.	Marula nut
Arecaceae	*Borassus akeassii* Bayton, Ouédr. & Guinko	Palmyra palm
Arecaceae	*Hyphaene thebaica* L.	Doum palm
Bombacaceae	*Adansonia digitata* L.	Baobab
Casuarinaceae	*Casuarina equisetifolia* L.	Australian pine tree
Combretaceae	*Guiera senegalensis* J.F. Gmel*.*	Moshi medicine
Fabaceae—Cesalpinaceae	*Cordyla pinnata* Lepr. ex A. Rich.	Bush mango
	*Piliostigma reticulatum* (DC) Hochst	Camel’s foot
	*Tamarindus indica* L.	Tamarind
Fabaceae–Mimosaceae	*Acacia holosericea* Cunn. ex G. Don	Fish poison/soapy wattle
	*Acacia tumida* F. Muell. ex Benth*.*	Pindan wattle
	*Albizzia lebbeck* (L.) Benth.	Koko
	*Faidherbia albida* Del.	Winter thorn
	*Parkia biglobosa* Jacq.	*Néré,* African locust tree
	*Prosopis africana* (Guill., Perrott. & Rich.) Taub.	Kirya, Ayan
Fabaceae–Papilionoideae	*Pterocarpus lucens* Lepr. ex Guill. et Perrott.	Barwood
Meliaceae	*Azadirachta indica* A. Juss.	Neem, Indian lilac
Sapotaceae	*Vitellaria paradoxa* C.F.Gaertn. (ex *Butyrospermum*)	*Karité*, Shea nut tree
Zygophyllaceae	*Balanites aegyptiaca* Del*.*	Desert date

### Data analysis

The dataset consisted of 314 entries (155 for agroforestry; 159 for RW) including information on treatments, rainfall, woody species, and crop yields (grain and biomass). When available, we recorded data on soil carbon and organic matter, soil nutrient availability, soil hydrological properties, and soil biological properties. The results were presented and discussed in light of relative effect size or response ratio (RR), calculated as the natural log (ln) difference between treatment and control (Eq. 1).

$$ \mathrm{RR}=\ln \left(\frac{\mathrm{treatment}\ \mathrm{yield}}{\mathrm{control}\ \mathrm{yield}}\right) $$

An RR above zero denotes beneficial effects of treatment over control conditions.

## Results and discussion

Sudano-Sahelian landscapes include a variety of perennial woody species that grow spontaneously in farmed agroforestry parklands and provide different forms of organic material useful for soil amendment: (a) when this vegetation is coppiced, fresh branches and leaves are used either as surface mulching material or slightly buried (Louppe 1991; Iyamuremye et al. 2000; Yélémou et al. 2007; Diack et al. 2010; Lahmar et al. 2012) and commonly combined with manure, crop residues, and/or compost prior to the growing season (Zongo 2009; Kabré 2010; Cabral 2011). Tree or shrub litter can be deposited in situ or transported (ex situ) to neighbouring fields that require organic biomass input. Alternatively, (b) when that vegetation is (partly) burned, farmers can incorporate the resulting ashes into the soil (Lufafa et al. 2008a; Lufafa et al. 2009).

Various structures of agroforestry systems may be identified in SWA, including parklands, fallows, hedgerows, and alley cropping (Fig. 3). Although not specifically captured through our literature retrieval strings, the Farmer Managed Natural Regeneration (FMNR—or *Regénération Naturelle Assistée*) approach stands out in both the published and grey literature from West Africa as an agroforestry strategy based on higher tree and shrub densities per hectare than traditional parklands. FMNR entails intensive management of shrubs, pruning and coppicing by farmers, in order to achieve optimal synergies between crops and trees on farmland. There is considerable literature, notably by ICRAF and others on FMNR initiatives in the Sahel, claiming that farmers adopting FMNR since the 1990s have “re-greened” millions of hectares through increases in tree/shrub densities (Weston et al. 2015). It is therefore necessary to highlight that FMNR differs from more traditional, sparse-tree-density forms of parkland agroforestry. Yet most cases in our literature retrieval refer to traditional parkland agroforestry systems, and thus further distinction between these and FMNR was not possible in our analysis.

The influence of trees on crop yields and soil characteristics in parkland farming systems of SWA has been documented extensively (Fig. 4b), with largely positive effects on average (Sanou et al. 2011; Bayala et al. 2012; Sanou et al. 2012; Coulibaly et al. 2013; Sinare and Gordon 2015). Crop yield is a classical farmer indicator to assess the success of agricultural innovations, which is often documented in research papers as well. In Section 3.1, we analyse effects of woody perennials on economic (grain) and biological (biomass) yields, followed by effects on soil properties (carbon, nutrients, water, and biology). Management options and resource limitations around the use of woody perennials in SWA are discussed in Sections 3.2 and 3.3, respectively.

### Crop yields and soil properties under parklands

#### Crop yields

**Fig. 5:**
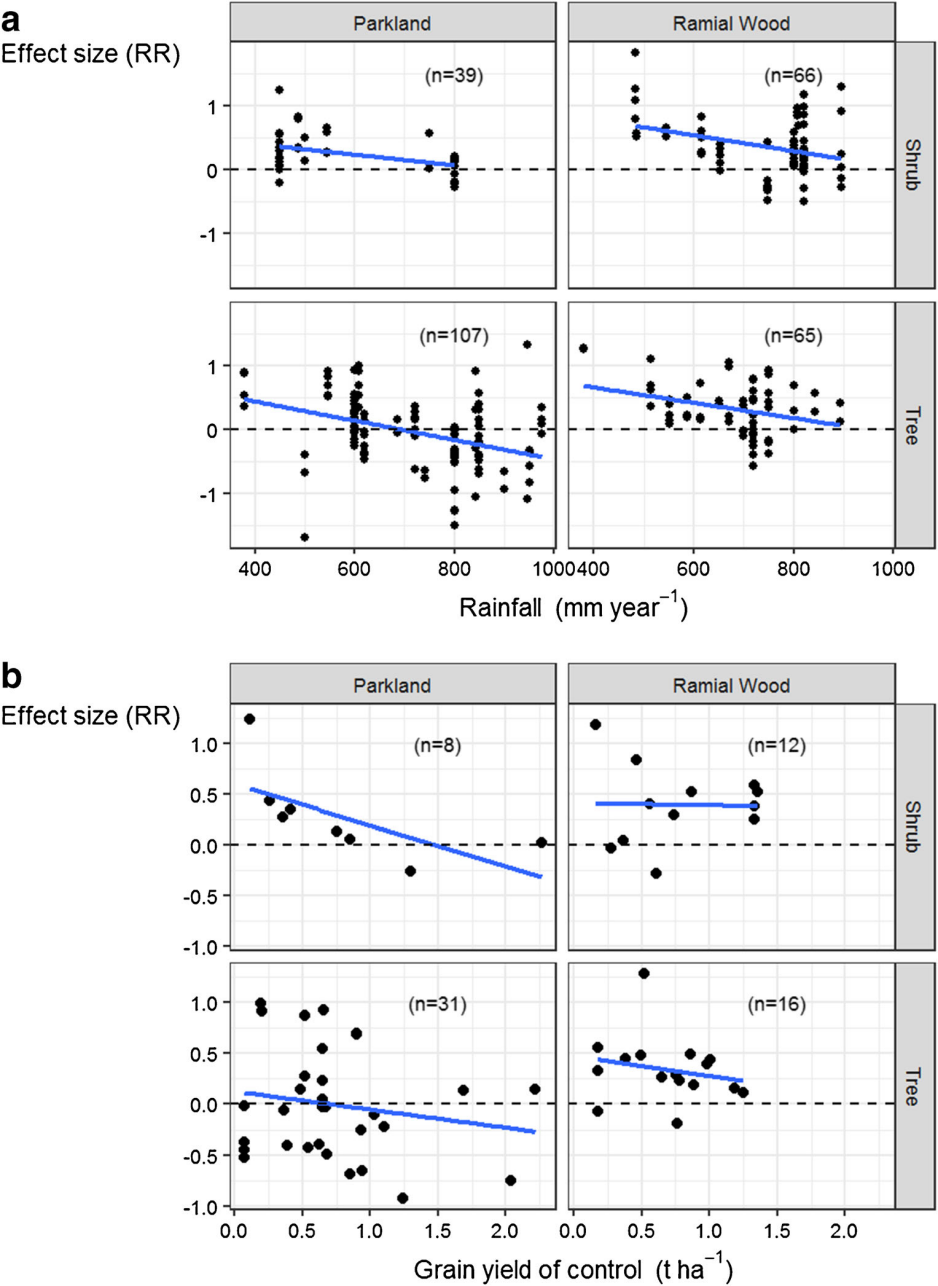
Effect size (response ratio) on yield over a rainfall gradient for all crops recorded in our meta-analysis (**a**) and effect size on millet and sorghum grain yields with treatments in function of the control grain yields (**b**)

**Fig. 6:**
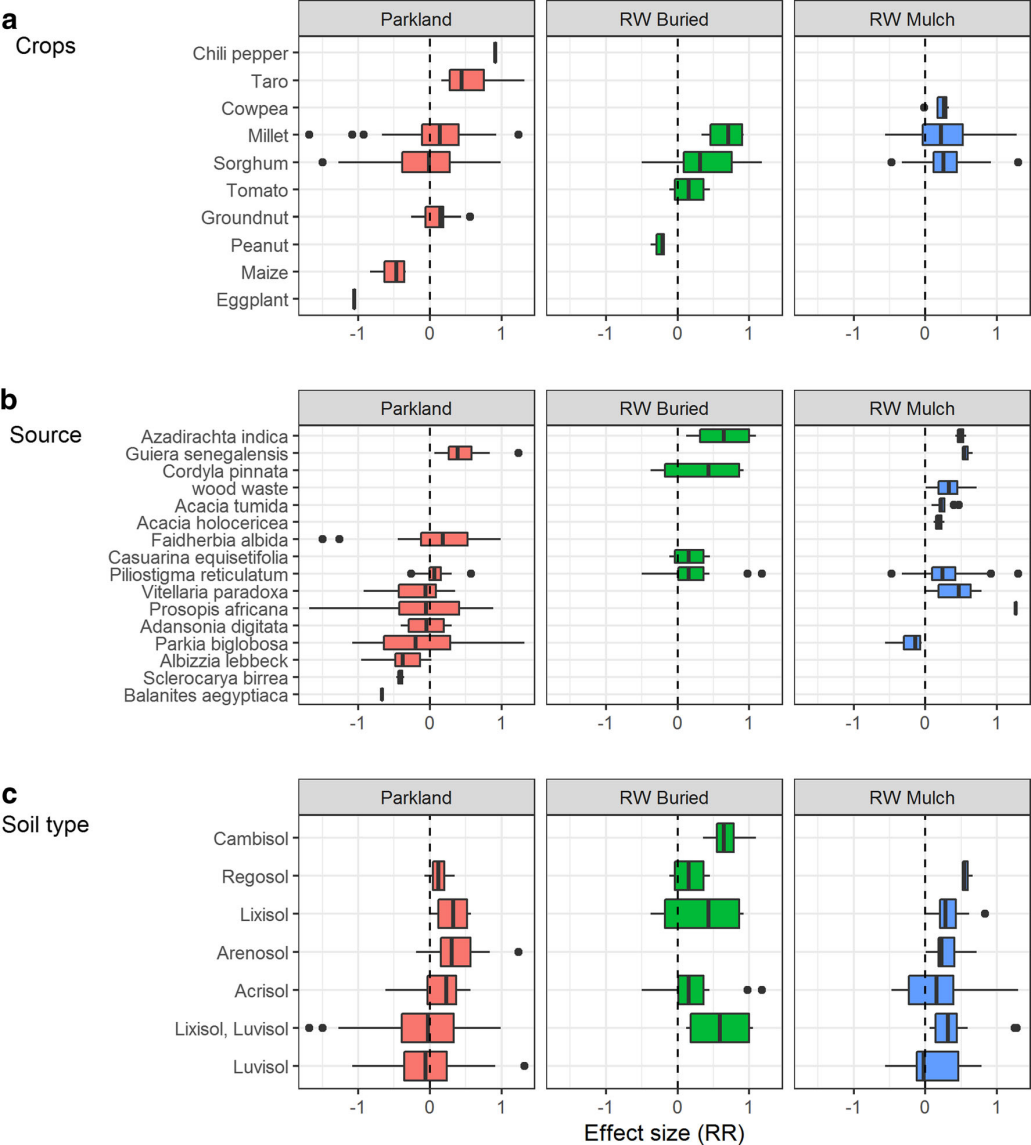
Effect size (RR) of yields per crop studied (**a**), per woody resource used (**b**), and per soil type (**c**)

The phenomenon of *resource islands*, or *fertility hotspots*, has been documented with the use of *P. reticulatum* shrubs as nursing trees for young mango trees, which increased chances of fruit production in semi-arid to arid conditions in Senegal (Hernandez et al. 2015). In a study from Niger, average grain yields reported in the vicinity of *G. senegalensis* shrubs were 773 kg ha^−1^ whereas yields in open fields (1.2-m radius from shrub) were only 382 kg ha^−1^ (Wezel 2000). Yield responses of sorghum and millet in parklands or with application of RW seem to be more important at low-rainfall sites, an effect that decreases with increasing rainfall (Fig. 5a). When environmental conditions for crop growth are not favourable (i.e. control yields are low), then sorghum and millet grain yields are less frequently affected next to the canopy of shrubs than under the canopy of trees (Fig. 5b). Overall, crops performed best when grown nearby *F. albida* and in the vicinity of shrubs such as *G. senegalensis* and *P. reticulatum*. Crops grown in the vicinity of species such as *Sclerocarya birrea*, *Albizzia lebbeck*, and *Balanites aegyptiaca* obtained less yields than those grown without the influence of these perennials (Fig. 6b).

In the vicinity of trees, taro and chili pepper performed better than maize and eggplant (Fig. 6a). Early studies on the effect of *F. albida* on groundnut productivity show no significant differences between the presence and the absence of trees (Louppe et al. 1996). Data from a long-term experiment in Senegal show that groundnut response is not significantly different when intercropped with shrubs (*G. senegalensis*) as compared to no shrubs (Dossa et al. 2013). Millet yields, however, increased in the presence of shrubs from the third to the 11th year of intercropping in the same trial (Bright et al. 2017).

In the case of *V. paradoxa*, Bazié et al. (2012) showed that chemical fertilisation on millet yields grown under the canopy of a tree would only show significantly higher results when the canopy of the tree was partially or totally pruned as compared to unpruned trees. Radiation is a major limiting factor to crop growth and development. When the crown is selectively pruned, then more light may be intercepted by crops growing underneath tree canopies. Pruning of the tree crown is particularly beneficial to cereals (C_4_ crops), yet growing shade-tolerant C_3_ crops (i.e. taro) may help lift the labour constraint of pruning the trees (Pouliot et al. 2011).

Coppiced material from woody perennials applied as mulch or buried ramial wood (RW) amendments had in most cases a positive effect on crop yields. As rainfall in the study area increased, the effect size of RW on crop yields decreased (Fig. 5a). In more than 70% of the cases, the yield responses were positive for cereal grain with the use of RW (Fig. 5b). Overall positive effects were noticeable for all crops (except peanut under RW buried; see Fig. 6a). Beneficial effects were observed for any given source of woody material employed as RW (except for *P. biglobosa*; see Fig. 6b).

Bayala et al. (2003) found that applying mulch of N-rich *P. biglobosa* leaves reduced millet grain and biomass yields while the N-rich leaf material of *V. paradoxa* increased millet yield as compared to control conditions. This effect was linked to the recalcitrant nature of the lignin (lig) and cellulose (cellu) composition of *P. biglobosa* leaves (C:N of 22, lig:cellu of 1.28) as compared to *V. paradoxa* leaves (C:N of 31, lig:cellu of 0.82)*.*

*P. reticulatum* leaf biomass applied as a mulch at 1.25 or 2.25 t DM ha^−1^ on a tropical ferruginous soil (Lixisol) in Burkina Faso increased sorghum grain yields by 14 and 28%, respectively, compared to a non-mulched control treatment (Yélémou et al. 2014). A study in semi-arid Niger reported that applying 2 t DM ha^−1^ of *G. senegalensis* mulch during two consecutive years resulted in increased millet grain yields by 76% during the first year and by 94% during the second year as compared to the non-mulch control (Wezel and Böcker 1999). Another experiment conducted on sandy-loam soils in Burkina Faso showed that yields decreased over the years on continuously cultivated plots, declining drastically during the second and third year for all treatments and control (Barthès et al. 2015). In that experiment, the use of 1.5 t DM ha^−1^ of *P. reticulatum* leaf and branch material as mulch did not significantly increase sorghum yields as compared to the control. The addition of RW does not automatically translate into increased crop yields, but the effects of RW use are rarely negative on crops (Figs. 5 and 6).

#### Soil carbon

**Fig. 7:**
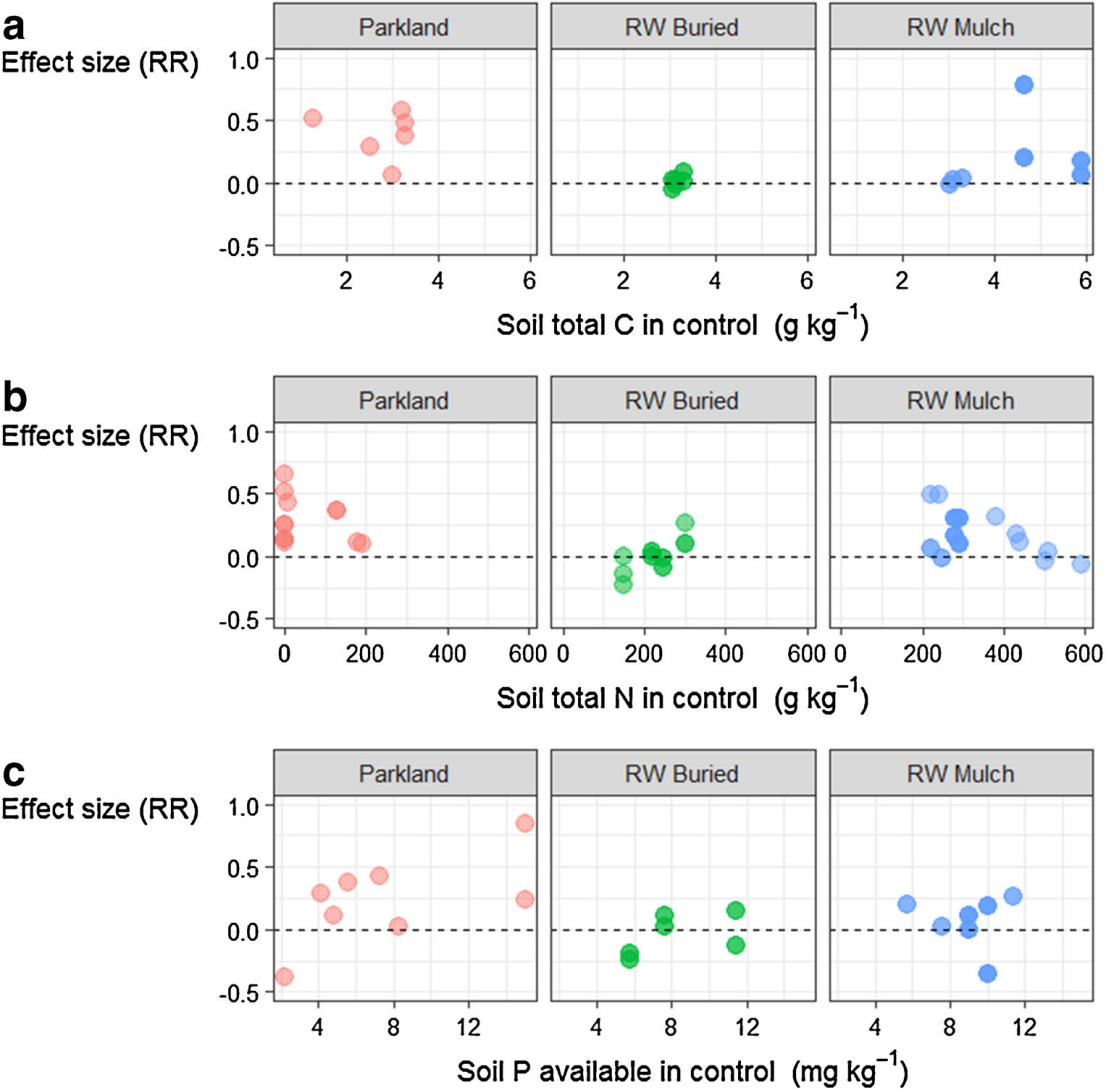
Effect size (RR) of treatments considered as a function of the value in control for soil total carbon (**a**), total nitrogen (**b**), and phosphorus (**c**) available

Higher total soil C content (+ 20 to 75%) under canopies of woody perennials (Fig. 7a) is normally ascribed to litter deposition and sediment trapping. “Fertility” islands under perennial shrub canopies are also linked to fine root decay (Manlay et al. 2004) and effective entrapment of wind-blown sediments (Wezel et al. 2000; Leenders et al. 2007), with sediment capture efficiency as a function of shrub density and canopy size (Mudrak et al. 2014). This effect may also be related to the presence (and decomposition) of herbaceous plants whose growth is favoured underneath the shrub crown (Yélémou et al. 2012). The presence of perennial species in agricultural fields may also create a microclimate due to shading and deposition of above- and belowground organic material, resulting in pedospheres with increased capacity for C sequestration. Measurements of soil δ^13^C isotopic content in Burkina Faso showed that soil C in the vicinity of tree trunks was mainly from tree origin (C_3_) and that C_4_-derived soil C (from cereals) was similar in the open field and nearby the perennials (Bayala et al. 2006).

In the vicinity of woody perennials, there is both decomposed and non-decomposed organic material (including fine roots). This enhances soil moisture retention and reduces evaporation. Additionally, when livestock consume parts of a shrub plant (e.g. *P. reticulatum* fruits) or rest under the shade of woody perennials, nutrients tend to be concentrated via livestock depositions. Moreover, migratory and resident birds find refuge and food in perennial vegetation of agricultural zones of the Sahel (Stoate et al. 2001) very likely further enriching soils in the vicinity of trees (i.e. *F. albida*) through deposition of organic droppings (Jonsson et al. 1999; Sileshi 2016).

Literature on soil C dynamics in response to woody biomass amendments reports different results. On the one hand, a study testing *P. reticulatum* shrub material as soil amendment in an experimental station reported slight (and non-significant) increases in soil C of less than 2% as compared to non-mulched conditions, and significantly lower with N addition, after 3 years (Barthès et al. 2015). On the other hand, a study on the use of *Cordyla pinnata* at a rate of 156 kg of mulch per t of soil reported extremely high increases of soil C of + 600% in a timeframe of 120 days as compared to no mulch (Samba 2001).

Soils amended with coppiced material as mulch tended to result in higher C contents than control soils that did not receive organic amendment, and higher than buried RW (Fig. 7a). As reported in the study of Bayala et al. (2003), *V. paradoxa* prunings increased soil C contents by 12% as compared to no-mulch conditions, while soils amended with *P. biglobosa* prunings enhanced soil C by 70% on average.

#### Soil nutrients

Soil total N and available P contents increased in the presence of woody perennials over situations without perennials, a probable indication of increased N and P deposition through litter and dust trapping, and potentially also in the case of N through some enhanced N_2_ fixation and/or enhanced conditions for N retention in soil around these vegetation structures (Fig. 7b, c). Soil N and P concentrations underneath scattered shrubs in semi-arid Niger were indeed 38 and 51% higher than in open fields, respectively (Wezel et al. 2000). In a study in Burkina Faso, total N was generally higher under *P. reticulatum* and *P. thonningii* than outside the shrub influence area (Yélémou et al. 2012), an effect observed in Niger as well, where soil N content was significantly higher in the vicinity of shrubs compared to zones more than 2 m away from the shrubs (Wezel 2000). This effect may be explained by greater nutrient input from organic material deposition, which itself can induce enhanced conditions for nitrifying bacteria. In a study in Senegal, soil total N, ammonium (NH_4_^+^), and total P contents in non-cropped areas were higher under canopies of *P. reticulatum* shrubs than on soils without shrubs, pointing towards enhanced nitrification processes nearby the perennials (Diakhaté et al. 2013).

Application of RW mulch had beneficial effects on soil N overall, but this effect was not so clear for RW buried (Fig. 7b). *P. biglobosa*, *V. paradoxa*, and *G. senegalensis* contributed 25 to 50% on average to soil N contents as compared to control treatments. *P. biglobosa* release of allelopathic components or lignin-to-N ratios may influence nutrient mineralisation rates (Bayala et al. 2005). Choice of species to use as RW amendments should be further explored in the light of C:N and lignin contents and the relation to N-immobilisation, particularly when amendments are buried. Effects of RW on available P followed a similar trend as for soil N (Fig. 7c).

#### Soil water

Systems with intercropped perennials show higher soil water content, making water more available for crop uptake. These results are largely due to belowground interactions, including (1) between roots and increased water infiltration that create preferential flows into deep layers of the soil (Bargues Tobella et al. 2014), (2) a reduction in soil evaporation under tree canopies (Jonsson et al. 1999), and (3) the hydraulic lift effect transporting deep-water and rewetting of surface soil water content, usually overnight (Bayala et al. 2008; Kizito et al. 2012).

In Burkina Faso, crops grown under *P. biglobosa* benefited from a 24% increase in soil water content as compared to control conditions (Wilson et al. 1998). In Senegal, soil water content was higher by 20 and 28% in millet cropping systems with *P. reticulatum* and *G. senegalensis*, under shrub crown (i.e. soils were moister) (Kizito et al. 2007). In this last study using neutron probes, the water balance for *P. reticulatum* and *G. senegalensis* shrubs intercropped with millet was superior to open-field observations, a difference explained by “hydraulic lift”. This phenomenon implies that water is transferred from deeper subsoil layers to the surface by deep-rooted perennials, and contributing to rewetting of upper soil horizons in the case of shrubs *P. reticulatum* and *G. senegalensis* (Kizito et al. 2012) and trees *P. biglobosa* and *V. paradoxa* (Bayala et al. 2008).

Enhanced infiltration rates may also result in higher water retention and storage in the upper layers of soil profiles underneath the canopy of the perennial species (Bargues Tobella et al. 2014). Woody and herbaceous species co-exist in semi-arid landscapes and differences in root proliferation zones may be such that different vegetation components do not seem to compete for water resources (Seghieri 1995).

*V. paradoxa* and *P. biglobosa* leaf applications as mulch in millet-based cropping systems were reported to decrease water ponding time in Burkina Faso (Bayala et al. 2003), with more pronounced effects under *V. paradoxa* mulch than under that of *P. biglobosa*. RW applications increase soil organic matter content which likely result in enhanced soil water contents (Chiroma et al. 2006). Moreover, organic matter additions will trigger termite activity, eventually leading to increased infiltration capacity (Mando 1997b; Mando and Miedema 1997; Ouédraogo et al. 2004).

#### Soil biology

Nutrient concentration in termite mounds as compared to that in adjacent soils may have both a biological origin from metabolic processes (saliva, faeces, plant debris) and a mineral origin from clay accumulation within nest structures (Sileshi et al. 2010). Termite-mediated processes increased nutrient recycling, promoted soil formation, and enhanced soil moisture retention and infiltration in several trials conducted on drylands (Mando 1997a, b; Mando and Miedema 1997; Mando 1998; Léonard and Rajot 2001; Laguemvare 2003; Mando and Stroosnijder 2006; Ouédraogo et al. 2007). Some of the species of Termitidae in semi-arid Burkina Faso include *Odontotermes smeathmani*, *Microtermes lepidus*, and *Macrotermes bellicosus* (Ouédraogo et al. 2004). Their diets include dry-wood, damp-wood, litter, and grass (Ouédraogo et al. 2007; Kaiser et al. 2015). There is a general consensus that termite foraging activity in SWA improves crop rainfall use efficiency when soils are mulched with either crop residues or ramial wood or with a combination of both (Léonard and Rajot 2001; Brussaard et al. 2007; Sileshi et al. 2010). Water infiltration may be reduced in the presence of subterranean termites (*Odontotermes* genus) because water-repellent particles such as organic matter and silt are being transported towards the surface layer, making it more impermeable locally (Mettrop et al. 2013). Termites in African savannahs contribute greatly to landscape heterogeneity (Davies et al. 2016), but further study is required to understand the effect of particular termite species on soil nutrient dynamics at a landscape scale and their influence on the spatial distribution of perennials (Kaiser et al. 2015).

Higher microbial biomass activity may support a wider range of biogeochemical processes through faster decomposition rates of catabolites within the rhizosphere of *P. reticulatum*-millet intercrops (Diakhaté et al. 2016). Yélémou et al. (2013) found that microbial respiration increased by 13 and 266% under the canopies of *P. reticulatum* and *P. thonningii* in agriculural fields. Diedhiou et al. (2009) showed that soil microbial communities from “resource islands” exhibited higher biomass and greater diversity, and exhibited much more fungal flora as compared to outside of the woody perennials’ area of influence (*G. senegalensis* and *P. reticulatum*). Their explanation of the process alludes to lower water stress and increased stimulation for litter decomposition processes. Diedhiou-Sall et al. (2013) studied, in controlled conditions, the effects of a composed substrate of *P. reticulatum* leaves and stems on soil microorganisms of Senegal that had never received woody amendments. The organic substrate input increased microbial biomass, as well as the levels of cellulase and β-glucosidase enzyme activities, which are closely related to C-mineralisation.

Hernandez et al. (2015) confirmed observations regarding the abundance, diversity, and higher activity of microorganisms within *P. reticulatum* resource islands in Senegal. Other studies have described microbial communities by focusing on the densities of predators, i.e. bacterivores, fungivores, and others. The presence of shrubs in Senegal increased the abundance of bacteria-feeding nematodes over that of plant-feeding nematodes (Diakhaté et al. 2013). This has important implications on nutrient cycling and availability since *P. reticulatum* influence tends to increase bacterivores and decrease plant-feeding nematodes (Diakhaté et al. 2013).

### Management options

The original forest landscape of SWA has been gradually cleared for crop cultivation at intensities that vary across the region (Fig. 1 and Fig. 2). The cutting of native trees and overstocking of transhumant cattle have contributed to desertification and degradation of fragile soils (Dongmo et al. 2012). Farming families in SWA have developed and/or adopted erosion and desertification control practices since the 1970s in order to counter drought (Critchley et al. 1994; West et al. 2008). This resulted in halting land degradation in SWA according to some views, which recognise a recent re-greening of the region thanks to soil restoration techniques (Mazzucato and Niemeijer 1998; Niemeijer and Mazzucato 2002; Farage et al. 2007; van Walsum et al. 2014). Tree-crop integrated systems, a form of agroforestry, occupy an increasingly important place in the development of sustainable farming systems in SWA (Gijsbers et al. 1994; Ouédraogo and Alexandre 1994; Yaméogo et al. 2013). These systems have been shown to be resilient and adaptive despite the ecological and economic crises that have affected Sudano-Sahelian agriculture in the past four decades (de Ridder et al. 2004; Aune and Bationo 2008; Reij and Smaling 2008; Hien et al. 2010; Settle and Garba 2011).

While woody perennials seem clearly linked to soil fertility hotspots, Bayala et al. (2015) advance that farming families would benefit more from the presence of trees on parklands by either (a) pruning dense tree canopies to minimise light competition with cereals or (b) maintaining the canopy but replacing cereals with shade-tolerant-species (i.e. taro). Identifying crop species and selection of woody perennials to associate with cereals will require mobilisation of ecological knowledge. Planting patterns that take into account environmental constraints (i.e. taro under *V. paradoxa* crown and cereals in the open fields) may further result in increased ecosystem service provision (i.e. biodiversity and improved household nutrition). Livestock breeders may also see yield benefits derived from tree- or shrub-based fodder as a complement to crop residues for animal nutrition. Woody perennials may furthermore provide renewable resources for fuelwood, construction material, and medicine (Yélémou et al. 2007; Sop et al. 2012). Applying FMNR is a low-investments method that can reconcile food production with reforestation and could furthermore improve economic and nutritional benefits from agriculture, including the improvement of health and psycho-social conditions (Weston et al. 2015).

### Resource limitations

Woody perennials prevailing in SWA tend to produce organic matter in the form of branches and leaves at relatively high rates despite being coppiced on a frequent basis. To illustrate woody and leafy biomass availability for soil amendments in a Sudano-Sahelian landscape, let us examine the example of a study led in Guié, a 47-km^2^ village of Centre-North Burkina Faso (Kabré 2010). In this locality, only six species were responsible for 63% of the stems sampled in a systematic forestry inventory (Table 5). *Acacia macrostachya* was the most abundant species, representing 17% of the sampled stems, yet this species has thorns and yields little leaf biomass. Therefore, when it occurs on the fields, it is usually slashed and burnt to ashes by farmers to clear cropping fields prior to the rainy season. *V. paradoxa* or shea butter tree, on the other hand, is abundant and accounts for higher biomass rates. Nevertheless, from the 829 kg DM ha^−1^ assessed in the study presented in Table 5, the biomass attributed to shea butter trees would be inaccessible as a source of soil amendments. This particular species is regulated in local and national policies since its products are consumed in local cuisine and exported as raw material for the international cosmetic industry. *V. paradoxa* is deciduous. The leaf biomass is often burnt while it could be composted or used directly as mulch. As a result, the remaining “available” woody and leafy biomass to apply on soils at Guié, Burkina Faso, includes three shrubs (*Combretum micranthum*, *G. senegalensis*, and *P. reticulatum*) and one tree species (*Cassia sieberiana*), accounting for less than 250 kg DM ha^−1^ (Kabré 2010).

**Table 5:** Potential woody biomass of three trees and three shrubs on a village territory of 47 km^2^ in Guié, Centre-North Burkina Faso. Data source: Kabré (2010)

Species	Woody plant type	% stems sampled	Woody branches biomass sampled < 7 cm (kg DM ha^−1^)	Woody twigs biomass sampled < 2 cm (kg DM ha^−1^)	Total woody biomass sampled (kg DM ha^−1^)
*Acacia machrostachya*	Tree	17	nd	nd	nd
*Combretum micranthum*	Shrub	13	32	18	50
*Cassia sieberiana*	Tree	13	61	38	99
*Vitellaria paradoxa*	Tree	11	356	290	646
*Guiera senegalensis*	Shrub	5	8	6	14
*Piliostigma reticulatum*	Shrub	4	10	10	20
Total		63	467	362	829

*nd* no data available

In Senegal, *P. reticulatum* accounted for ca. 1 t ha^−1^ while *G. senegalensis* for 1–2 t ha^−1^ of C-rich biomass (Lufafa et al. 2008b). Earlier studies in Senegal measured no more than 500 kg DM ha^−1^ of *G. senegalensis* (Louppe 1991). These are rather low amounts as compared to the 1–6 t DM ha^−1^ that is applied in experiments documented in scientific literature. It is essential to take into account that the feasibility of RW use depends on:

1. Effect of soil amendments on yield, nutrient cycling, and soil C-sequestration rates
2. Richness and composition of woody perennials in the ecosystem
3. Frequency of pruning
4. Proximity between *source* and *sink* sites

The intrinsic capacity of a given species to re-sprout vigorously and produce a large biomass is a trait that is mediated by the environment and management. Identifying species with such trait is key and might be one of the future investigation areas. Transferring leaves and branches over long distances to apply on degraded soils represents an appreciable workload that may conflict with other on-farm or off-farm labour demands. Attention must also be given to local land tenure rules on the use, management, and plantation of trees and shrubs on agricultural fields, which may sometimes be disconnected from those promoted by institutions (Rousseau et al. 2017).

## Conclusion

The objective of this meta-analysis was to qualify and quantify the effects that woody perennials have on crop productivity and soil characteristics. We specifically studied the effects of woody perennials in agroforestry systems and the effects of coppiced material application as soil amendments (i.e. ramial wood, RW). Woody perennials in agroforestry systems locally create resource islands or fertility hotspots around their base, related to both aboveground (i.e. litter addition) and underground (i.e. hydraulic lift and root decay) processes. From this meta-analysis, it is possible to advance that shrubs in cereal-based cropping systems render similar benefits as trees (i.e. enhanced soil properties) with fewer trade-offs in terms of yield. These effects were more visible with low rainfall and at low site productivity.

RW amendments had overall beneficial effects on crop yields and on soil C and N contents, independent of source material. Considerations on the resource availability and sustainability of the practice need to be further studied. Application of RW material from shrubs and trees may be an option to improve agricultural soils of semi-arid West Africa in perennial-annual crop systems. The results of this meta-analysis highlight the need for research on the use and management of perennials (especially of shrubs), on the synergies that occur amongst biodiversity components at the cropping system level, and on the trade-offs at the farming system level.

In the light of discussions about “greening the Sahel” through tree planting, or the establishment of a “Great Green Wall in capital letters” south of the Sahara, our meta-analysis indicates that great potential in terms of increasing and stabilising soil productivity can be derived from the intensive management of existing native woody vegetation, in multifunctional landscapes that combine food crop production with other tree-mediated ecosystem services.
